# Potential Antioxidant Properties of Enzymatic Hydrolysates from *Stichopus* *japonicus* against Hydrogen Peroxide-Induced Oxidative Stress

**DOI:** 10.3390/antiox10010110

**Published:** 2021-01-14

**Authors:** Hyo-Geun Lee, Hyun-Soo Kim, Jae-Young Oh, Dae-Sung Lee, Hye-Won Yang, Min-Cheol Kang, Eun-A Kim, Nalae Kang, Junseong Kim, Soo-Jin Heo, You-Jin Jeon

**Affiliations:** 1 Department of Marine Life Sciences, Jeju National University, Jeju 63243, Korea; hyogeunlee92@gmail.com (H.-G.L.); koty221@naver.com (H.-W.Y.); 2 Marine Biodiversity Institute of Korea, Seocheon 33362, Korea; gustn783@mabik.re.kr (H.-S.K.); daesung@mabik.re.kr (D.-S.L.); 3 Food Safety and Processing Research Division, National Institute of Fisheries Science, Busan 46083, Korea; ojy0724@naver.com; 4 Research Group of Food Processing, Korea Food Research Institute, Wanju 55365, Korea; mckang@kfri.re.kr; 5 Jeju International Marine Science Center for Research & Education, Korea Institute of Ocean Science and Technology (KIOST), Jeju 63349, Korea; euna0718@kiost.ac.kr (E.-AK.); nalae1207@kiost.ac.kr (N.K.); junseong@kiost.ac.kr (J.K.)

**Keywords:** antioxidant, red sea cucumber, *Stichopus japonicus*, enzyme-assisted extract, oxidative stress

## Abstract

A comprehensive antioxidant evaluation was performed on enzymatic hydrolysates of *Stichopus* *japonicus* (*S. japonicus*) using Vero cells and zebrafish models for in vitro and in vivo studies, respectively. *S. japonicus* was hydrolyzed with food-grade enzymes (alcalase, α-chymotrypsin, flavourzyme, kojizyme, neutrase, papain, pepsin, protamex, and trypsin), and the free radical scavenging activities were screened via electron spin resonance (ESR) spectroscopy. According to the results, the enzymatic hydrolysates contained high protein and relatively low polysaccharide and sulfate contents. Among these hydrolysates, the α-chymotrypsin assisted hydrolysate from *S. japonicus* (α-chy) showed high yield and protein content, and strong hydroxyl radical scavenging activity. Therefore, α-chy was chosen for further purification. The α-chy was fractionated by ultrafiltration into three ultrafiltration (UF) fractions based on their molecular weight: >10 kDa (α-chy-I), 5–10 kDa (α-chy-II), and <5 kDa (α-chy-III), and we evaluated their antioxidant properties in H_2_O_2_ exposed Vero cells. The α-chy and its UF fractions significantly decreased the intracellular reactive oxygen species (ROS) generation and increased cell viability in H_2_O_2_ exposed Vero cells. Among them, α-chy-III effectively declined the intracellular ROS levels and increased cell viability and exhibited protection against H_2_O_2_ induced apoptotic damage. Furthermore, α-chy-III remarkably attenuated the cell death, intracellular ROS and lipid peroxidation in H_2_O_2_ exposed zebrafish embryos. Altogether, our findings demonstrated that α-chy and its α-chy-III from *S. japonicus* possess strong antioxidant activities that could be utilized as a bioactive ingredient for functional food industries.

## 1. Introduction

Oxidative stress is induced by excessive reactive oxygen species (ROS) generation [1]. In general, superoxide (O_2_^−^), hydrogen peroxide (H_2_O_2_), singlet oxygen (^1^O_2_), hypochlorous acid (HOCl), peroxyl radicals (ROO^•^), hydroperoxyl radicals (HOO^•^), and hydroxyl radicals (HO^•^) are generated as byproducts of aerobic metabolism in living organisms [2]. However, excessive or uncontrolled ROS production may result in oxidative stress, which is detrimental and leads to irreversible chemical modifications resulting in cell death, apoptosis, and the oxidation of cellular components [3]. Moreover, prolonged oxidative stress could be associated with the pathogenesis of human diseases such as cancer, inflammation, diabetes, and hypertension. However, oxidative damage could be ameliorated by natural or synthetic antioxidants used for medicinal purposes. However, synthetic antioxidants such as butylated hydroxyanisole and butylated hydroxytoluene exhibit toxicity after long-term use [4]. Consequently, many researchers have focused on natural antioxidant doses that do not show toxicity on usage. Marine organisms are a rich source of bioactive natural products, including polysaccharides, proteins, carotenoids, and polyphenolic compounds, which may have vulnerable biological properties including antioxidant, anticancer, anti-inflammatory, anti-diabetic, and anti-obesity activities [5]. Over the past few decades, large-scale studies have been conducted on marine-derived natural products. Among these products, protein hydrolysate is the most important functional material obtained from marine organisms. It has enormous potential as a functional material in the future [6,7]. Many researchers have studied the antioxidant activities of protein hydrolysates [8,9,10].

*Stichopus japonicus* (*S. japonicus*) is a marine invertebrate native to coastal Korea, Japan, and China, and is used in food and folk medicine in Asian countries [11,12]. The Food and Agriculture Organization (FAO) of South Korea reported that *S. japonicus* is an industrially important species for Southeast Asian fisheries [13]. In previous studies, the enzymatic hydrolysate from *S. japonicus* possessed various biological properties, including antioxidant, antitumor, and anticoagulant activities [14,15,16,17].

However, the antioxidant activities of enzymatic hydrolysates of *S. japonicus* (SJH) have not been fully investigated. Therefore, in the present study, we investigated the comprehensive antioxidant properties of enzymatic hydrolysates from *S. japonicus* using Vero cells and zebrafish embryos for in vitro and in vivo studies, respectively.

## 2. Materials and Methods

### 2.1. Chemicals and Materials

Barium chloride dihydrate, ethanol, iron (II) sulfate heptahydrate, acetic acid, hydrochloric acid, nitric acid, ammonium sulfate, sodium hydroxide, phenol, and sulfuric acid were purchased from Daejung (Seoul, Korea). From Sigma-Aldrich (St. Louis, MO, USA), 2,2-Azino-bis (3-ethylbenzothiazoline-6-sulfonic acid) diammonium salt, 2,2-azobis (2-methylpropionamidine) dihydrochloride, 1,1-diphenyl-2-picrylhydrazyl (DPPH), gallic acid, glucose, Folin and Ciocalteu’s phenol reagent, peroxidase, α-(4-pyridyl N-oxide)-N-tert-butylnitrone, 5,5-dimethyl-1-pyrroline-N-oxide, 2′,7′-dichlorofluorescein diacetate (DCF-DA), 3-(4,5-dimethylthiazol-2-yl)-2,5-diphenyltetrazolium bromide (MTT), dimethyl sulfoxide (DMSO), and gum arabic were purchased. RPMI-1640 medium, fetal bovine serum, penicillin-streptomycin, and trypsin were purchased from Gibco (Mississauga, ON, Canada). Sodium carbonate anhydrous (Yakuri, Japan), bicinchoninic acid protein assay kit (Thermo Fisher Scientific, Pittsburgh, PA, USA), bovine serum albumin (Bovogen, East Keilor VIC, Australia) and hydrogen peroxide (Junsei, Tokyo, Japan) were used. Commercial food-grade enzymes (alcalase (Al), α-chymotrypsin (α-chy), flavourzyme (Fla), kojizyme (Koj), neutrase (Neu), papain (Pap), pepsin (Pep), protamex (Pro), and trypsin (Try)) were purchased from Novozyme (Bagsvaerd, Copenhagen, Denmark).

### 2.2. Preparation of the Enzymatic Hydrolysates from S. japonicus

*Stichopus japonicus* (*S. japonicus*) was kindly provided by the Korea Institute of Ocean Science and Technology. The intestine-removed *S. japonicus* was washed with tap water and dried. The dried *S. japonicus* was homogenized with a grinder and maintained at −20 °C. 

To examine the antioxidant effect of the enzymatic hydrolysates of *S. japonicus* (SJH), enzyme-assisted extraction was adopted. Enzymatic hydrolysis was performed using food-grade enzymes (Al, α-chy, Fla, Koji, Neu, Pap, Pep, Pro, and Try) under optimal conditions as described by Byun and Kim (2001) [18]. Briefly, 1 g of *S. japonicus* and 10 mg of enzyme were mixed with 100 mL of deionized water. The mixtures were then incubated in a shaking incubator for 24 h. After 24 h, the mixtures were clarified by centrifugation (8000 rpm, 4 °C, 10 min). The mixtures were filtered through Whatman paper, their pH was adjusted to 7.00, and they were freeze-dried for further experiments. 

### 2.3. Measurement of Yield and Proximate Composition

The yield and proximate composition of each SJH were investigated. The yields were calculated as the percentage of dry weight compared to the hydrolyzed sample weight. The total polysaccharide, protein, polyphenol and sulfate contents were measured using the phenol–sulfuric acid method [19], Lowry protein assay [20], Folin–Ciocalteu method [21], and barium–gelatin method [22], respectively.

### 2.4. Ultrafiltration and Molecular Distribution of SJH

To separate the peptide fraction, ultrafiltration (UF) was carried out on SJH. Separation was performed using a decreased molecular mass order from 10 kDa to 5 kDa. The SJH was fractionated using a UF device (Lab scale TFF system, Millipore, Burlington, MA, USA) equipped with a molecular weight cut-off (MWCO) membrane. It was passed through the largest 10 kDa MWCO UF membrane. The retentate (>10 kDa; α-chy-I) and the permeate (below 10 kDa) were collected, and the permeate was applied to a 5 kDa membrane to separate the retentate (5 to 10 kDa; α-chy-II) and the permeate (<5 kDa; α-chy-III) UF fractions. The obtained UF fractions were lyophilized and stored at −70 °C. Molecular distribution of UF fractions from the SJH was determined with sodium dodecyl sulfate-polyacrylamide gel electrophoresis (SDS-PAGE), which was carried out using 15% SDS separation gel and 4% stacking gel. The loading samples were heated at 100 °C for 3 min before electrophoresis. Electrophoresis was performed using a Mini-PROTEIN Tetra System (Bio-Rad, Hercules, CA, USA) at 80 V for 1–2 h.

### 2.5. Amino Acid Profile

Amino acid compositions were analyzed using an amino acid auto analyzer (S433-H, SYKAM, Eresing, Germany) as described by Asaduzzaman and Chun (2015) [23]. The samples were introduced to an LCA K06/Na cation separation column (4.6 × 150 mm) and eluted with 5 mM of p-toluenesulfonic acid solution as the mobile phase at a flow rate of 0.45 mL/min. The 5 mM of p-toluenesulfonic acid containing 100 mM ethylenediaminetetraacetic acid (EDTA) and 20 mM bis-tris was used as a post-column reagent at a 0.25 mL/min flow rate. The amino acids were detected using a fluorescence spectrophotometer at 440 nm and 570 nm.

### 2.6. Free Radical Scavenging Activity

The free radical scavenging activities were screened using electron spin resonance (ESR; JEOL, Akishima, Tokyo, Japan). The free radical scavenging activities were determined using modified methods involving DPPH, hydroxyl, and alkyl radicals [24,25].

### 2.7. Cell Line and Cell Culture

Monkey kidney fibroblasts (Vero cells) were purchased from the Korean Cell Line Bank. Vero cells were cultured in RPMI-1640 medium, supplemented with 10% fetal bovine serum, 1% streptomycin (100 μg/mL), and penicillin (100 unit/mL^−1^) and maintained at 37 °C in a 5% CO_2_ incubator [26].

### 2.8. Determination of Cell Viability and Intracellular ROS Generation in H_2_O_2_ Exposed Vero Cells

The potential antioxidant activities were evaluated under H_2_O_2_ induced oxidative conditions. Briefly, Vero cells were plated in 96-well plates at a concentration of 1 × 10^5^ cells/mL^−1^ and incubated for 24 h. After 24 h of incubation, the samples were treated before activating them with H_2_O_2_ (1 mM) for 1 h. Subsequently, 2 mg/mL of MTT solution was added and the cells were incubated for an additional 2–3 h. Cell viability was measured using the MTT assay [27]. The intracellular ROS scavenging activity was analyzed using the DCF-DA assay [28]. The Vero cells were seeded as before, treated with H_2_O_2_ and different concentrations of samples, and incubated for 24 h. After 24 h of incubation, 500 µg/mL of DCF-DA was added to each well. Finally, DCF-DA fluorescence was measured using a Synergy HT Multi-Detection microplate reader (BioTek Instruments, Winooski, VT, USA) at an excitation and emission wavelength of 485 nm and 535 nm, respectively.

### 2.9. Detection of Apoptosis Using Propidium Iodide/Hoechst 33342 Double Fluorescent Staining

Propidium iodide (PI) and Hoechst 33342 double staining was conducted to confirm the protective effect of α-chy-III against H_2_O_2_ induced apoptotic DNA damages. Propidium iodide (PI) and Hoechst 33342 staining were performed using the fluorescent staining method described by Agarwal et al. [29]. Briefly, Vero cells were seeded in a 24-well plate and treated with the samples before exposure to H_2_O_2_ (1 mM). After 12 h of incubation, the cells were stained with PI and Hoechst 33342, a DNA-specific fluorescent dye, for 10 min. The fluorescent images were observed under a fluorescence microscope equipped with a Cool SNAP-Pro color digital camera (Olympus, Tokyo, Japan).

### 2.10. Cell Cycle Analysis by Flow Cytometry

Flow cytometry was adopted to establish whether the protective effect of α-chy-III on H_2_O_2_ induced cell cycle arrest [30]. Vero cells were seeded as previously described. The cells were then treated with H_2_O_2_ (1 mM) before sample treatment and incubated for 24 h. After 24 h of incubation, the cells were harvested and fixed with 70% ethanol solution for 30 min at 4 °C. Then, the cells were washed with PBS three times by centrifugation (2000 rpm, 5 min). After washing, PI (10 µg/mL) staining was conducted for 30 min in darkness. After PI staining, the apoptotic sub-G1 contents were measured using a FACS-Calibur flow cytometer (Becton Dickinson, San Jose, CA, USA). The cell cycles were analyzed using the Quest and Mod-Fit (Verify Software, Topsham, ME, USA).

### 2.11. Origin and Maintenance of Parental Zebrafish

Adult zebrafish were purchased from a commercial dealer (Seoul Aquarium, Seoul, Korea), and 15 fish were housed in an acrylic tank under the following environmental conditions: 28.5 °C ± 1 °C, with a 14/10 h light/dark cycle. The zebrafish were fed twice daily, 6 days a week. Embryos were obtained from natural spawning that was induced in the morning by turning on the light. They were interbred using one female and two males. 

### 2.12. Treatment of Zebrafish Embryos with α-chy-III 

Approximately 7–9 h post-fertilization (hpf), the embryos (each group = 15 embryos) were transferred to the individual wells of 12-well plates and maintained in embryo medium. Then, different concentrations of α-chy-III (25, 50, 100, 200 μg/mL) were added 1 h before H_2_O_2_ (5 mM) treatment and incubated until 24 hpf. 

### 2.13. Measurement of Heart Rate and Survival Rate

The heart rate was measured at 2 days post fertilization (dpf) and the survival rate was determined at 3 dpf. The heart rate was recorded after treating the embryo with H_2_O_2_ for 30 s under the microscope. The result was calculated using the average heart rate per 30 s. The survival rate was calculated by counting the number of live zebrafish larva at 3 dpf. 

### 2.14. Measurement of Cell Death, Intracellular ROS, and Lipid Peroxidation in H_2_O_2_ Exposed Zebrafish Embryos

The effect of α-chy-III on cell death, intracellular ROS, and lipid peroxidation was investigated in H_2_O_2_ exposed zebrafish embryos according to the method from Kang et al. (2013) [31]. Here, 7–9 h post-fertilization (hpf) zebrafish embryos were transferred to 15 embryos as previously described. Then, they were co-treated with α-chy-III and H_2_O_2_ and incubated until 3 dpf. At 3 dpf, the embryos were anesthetized for fluorescent staining of cell death, intracellular ROS, and lipid peroxidation. The fluorescence was observed under a fluorescent microscope, which was equipped with a Cool SNAP-Pro color digital camera (Olympus, Japan), and individual fluorescence intensity was quantified using the ImageJ program.

### 2.15. Statistical Analysis

All measurements were presented as the mean ± standard error, and a one-way ANOVA test was performed. Significant differences between the means of parameters were determined using the Tukey’s post hoc comparison and Duncan’s multiple range test.

## 3. Results

### 3.1. The Yield, Chemical Composition, and Free Radical Scavenging Activities of Enzymatic Hydrolysates of S. japonicus

*Stichopus japonicus* (*S. japonicus*) was hydrolyzed with distilled water (DW) and nine enzymes (Al, α-chy, Fla, Koj, Neu, Pap, Pep, Pro, and Try). The extraction yield and chemical composition of the extracts are summarized in Table 1. All the enzymatic hydrolysates of *S. japonicus* (SJH) contained a high protein content and relatively low polysaccharide and sulfate contents. Among them, α-chymotrypsin assisted hydrolysate from *S. japonicus* (α-chy) showed a high extraction yield (96.50 ± 0.06%), containing the highest levels of protein (34.05 ± 0.97%) compared with the aqueous extract. In addition, the α-chy showed the highest hydroxyl radical scavenging activity compared to the other samples (Table 2).

### 3.2. Screening of the Potential Antioxidant Effect of SJH

**Figure 1:**
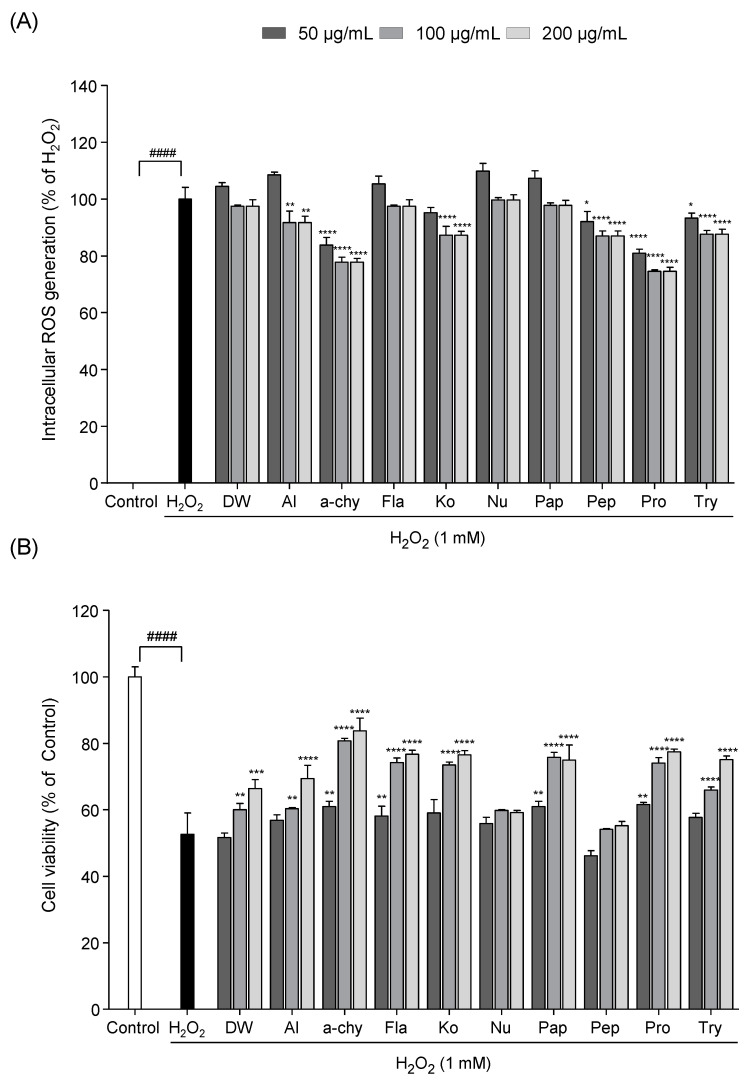
Protective effect of SJH against H_2_O_2_ induced oxidative stress. Intracellular reactive oxygen species (ROS) scavenging activity (**A**) and cell viability (**B**) in H_2_O_2_ exposed Vero cells. Experiments were performed in triplicate and data are expressed as mean ± SD; Significant differences identified at * *p* < 0.05, ** *p* < 0.01, *** *p* < 0.001 and **** *p* < 0.0001 as compared to the H_2_O_2_ treated group; ^####^ *p* < 0.0001 as compared to the control group. Statistical analyses were conducted using Tukey’s post hoc comparison and Duncan’s multiple range test.

To establish the free radical scavenging activity of SJH in vitro, intracellular ROS production and cell viability were measured in Vero cells exposed to H_2_O_2_. As shown in Figure 1, intracellular ROS and cell death were increased in H_2_O_2_ stimulated Vero cells. However, SJH markedly reduced ROS production and increased cell viability. Among them, α-chy showed a strong protective effect against H_2_O_2_ induced oxidative damage compared with the other samples.

### 3.3. Separation and Molecular Weight Distribution of α-chy and Its UF Fractions

**Figure 2:**
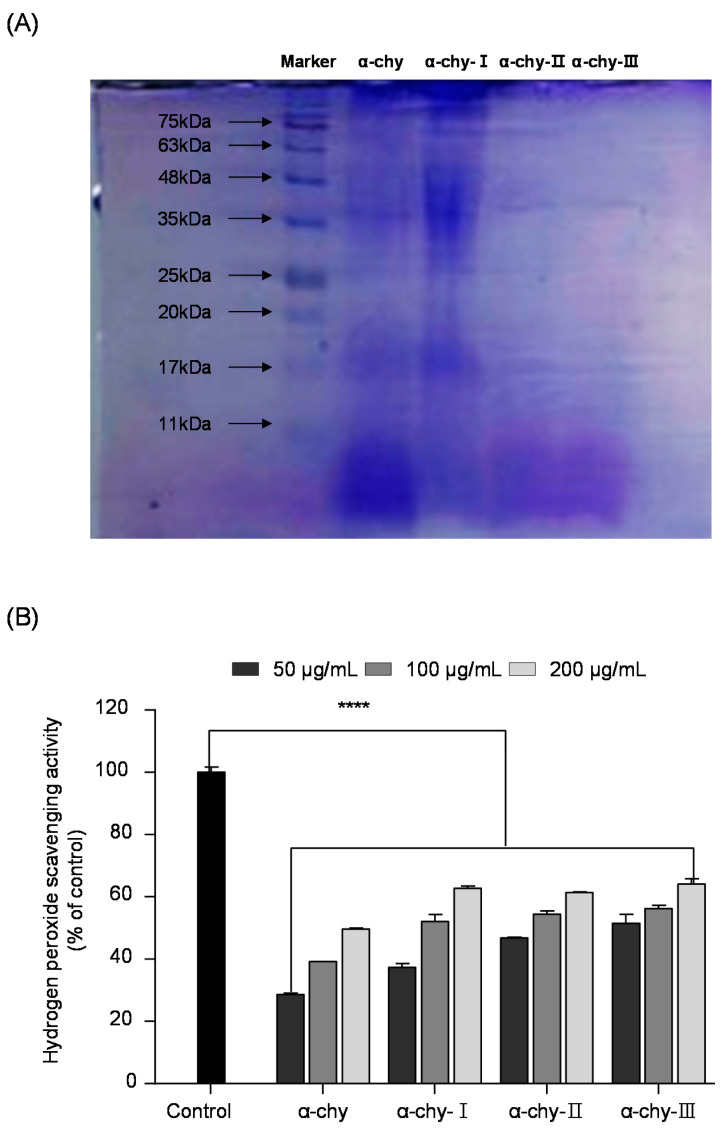
Molecular weight distribution and hydrogen peroxide scavenging activity of α-chy and its UF fractions. Sodium dodecyl sulfate-polyacrylamide gel electrophoresis (SDS-PAGE) pattern (**A**) and hydrogen peroxide scavenging activity of UF fractions (**B**). Experiments were performed in triplicate and data are expressed as mean ± SD; Significant difference identified at **** *p* < 0.0001 as compared to the control group.

The α-chy was separated with different molecular weight fractions via ultrafiltration (UF). As shown in Figure 2A, the molecular SDS-PAGE analysis indicated that the SJH was separated with three ranges of UF fractions (>10 kDa (α-chy-I), 5–10 kDa (α-chy-II), <5 kDa (α-chy-III)). We evaluated their antioxidant properties using hydrogen peroxide scavenging analysis. According to the results, all the UF fractions showed significant hydrogen peroxide scavenging activity, and the low molecular weight α-chy-III showed the highest hydrogen peroxide scavenging activity compared with the other UF fractions.

### 3.4. Amino Acid Profiles of α-chy and Its UF Fractions

The amino acid compositions of α-chy and its UF fractions are summarized in Table 3. The α-chy and its UF fractions were composed of seven essential amino acids (threonine, valine, methionine, leucine, tyrosine, phenylalanine, and histidine) and non-essential amino acids (aspartic acid, serine, glutamic acid, proline, glycine, alanine, isoleucine, lysine, and arginine). Commonly, the α-chy and its UF fractions consisted of high aspartic acid, glutamic acid, glycine, and arginine. In particular, α-chy-III contained high ratios of proline, glycine, methionine, tyrosine, phenylalanine, lysine, and arginine contents.

### 3.5. Effect of α-chy and Its UF Fractions against H_2_O_2_ Induced Oxidative Stress in Vero Cells

**Figure 3:**
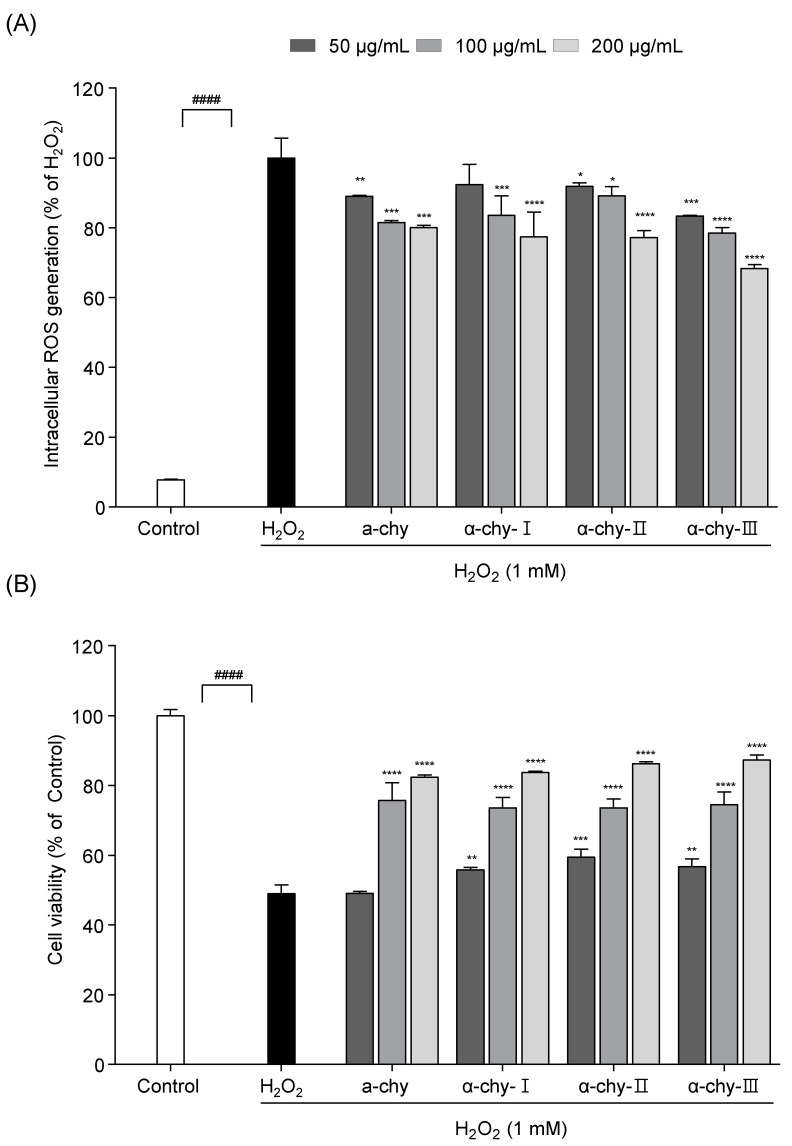
α-chy and its UF fractions suppress H_2_O_2_ induced oxidative damage in vitro Vero cells. Intracellular ROS scavenging activity (**A**) and cell viability (**B**) in H_2_O_2_ exposed Vero cells. Experiments were performed in triplicate and data are expressed as mean ± SD; Significant differences identified at * *p* < 0.05, ** *p* < 0.01, *** *p* < 0.001 and **** *p* < 0.0001 as compared to the H_2_O_2_ treated group; ^####^ *p* < 0.0001 as compared to the control group.

To evaluate the potential antioxidant effect of α-chy and its UF fractions, the MTT and DCF-DA assay were performed in H_2_O_2_ exposed Vero cells. As shown in Figure 3, a significant ROS generation and cell death were observed in the H_2_O_2_ treated group. However, the α-chy and its UF fractions markedly reduced the ROS and cell death levels. Among them, α-chy-III, which had the lowest molecular weight, showed the highest protection against H_2_O_2_ induced oxidative stress. α-chy-III (200 μg/mL) reduced ROS generation to 70% and increased cell viability by 80%.

### 3.6. Effect of α-chy-III against H_2_O_2_ Induced Apoptosis in Vero Cells

**Figure 4:**
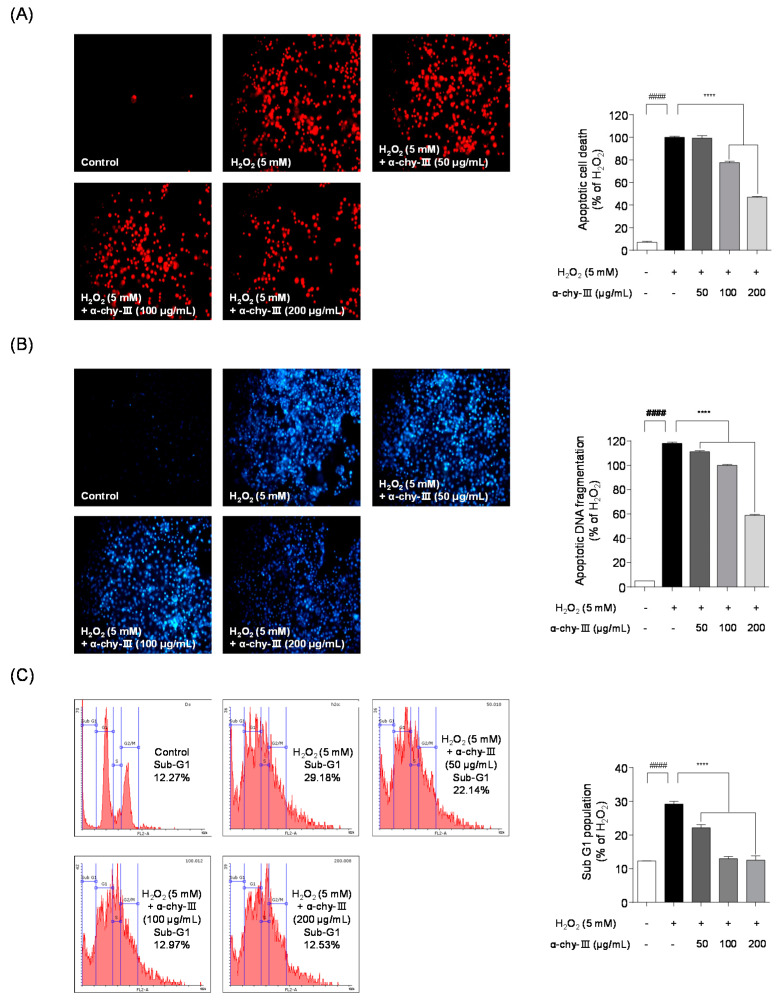
Effects of α-chy-III against H_2_O_2_ induced apoptotic cell death, DNA fragmentation and cell cycle regulation in Vero cells. Protective effect of α-chy-III in H_2_O_2_ induced apoptotic cell death (**A**) DNA fragmentation (**B**) and cell cycle regulation (**C**). The apoptotic cell death, DNA fragmentation and cell cycle regulation were analyzed via fluorescence microscopy before propidium iodide (PI) and hoechst 33342 staining. Experiments were performed in triplicate and data are expressed as mean ± SD; Significant differences identified at **** *p* < 0.0001 as compared to the H_2_O_2_ treated group; ^####^ *p* < 0.0001 as compared to the control group. Statistical analyses were conducted using Tukey’s post hoc comparison and Duncan’s multiple range test.

Next, we studied whether α-chy-III reduces H_2_O_2_ induced apoptotic damage, such as apoptosis, cell membrane destruction, cellular oxidation, and DNA fragmentation, using fluorescence staining. As shown in Figure 4, Hoechst 33342/PI staining results indicated significant apoptotic cell death and DNA fragmentation in the H_2_O_2_ treated group. Pretreatment with α-chy-III (200 μg/mL) significantly reduced apoptotic cell death and DNA fragmentation. These results suggest that α-chy-III significantly protects against H_2_O_2_ induced apoptotic damage in Vero cells. In addition to PI staining, flow cytometry was conducted to evaluate the protective effect of α-chy-III on H_2_O_2_ induced apoptotic cell cycle arrest. In the flow cytometry results, the apoptotic sub-G1 increased in the H_2_O_2_ treated group (29.18 ± 0.81%) compared to that in the control group (12.27 ± 0.02%). However, the sub-G1 population was significantly lowered following α-chy-III administration. These results suggest that α-chy-III protects against H_2_O_2_ induced apoptotic cell cycle arrest in Vero cells.

### 3.7. Effect of α-chy-III against H_2_O_2_ Induced Cell Death, ROS Generation, and Lipid Peroxidation in Zebrafish Enbryos 

**Figure 5:**
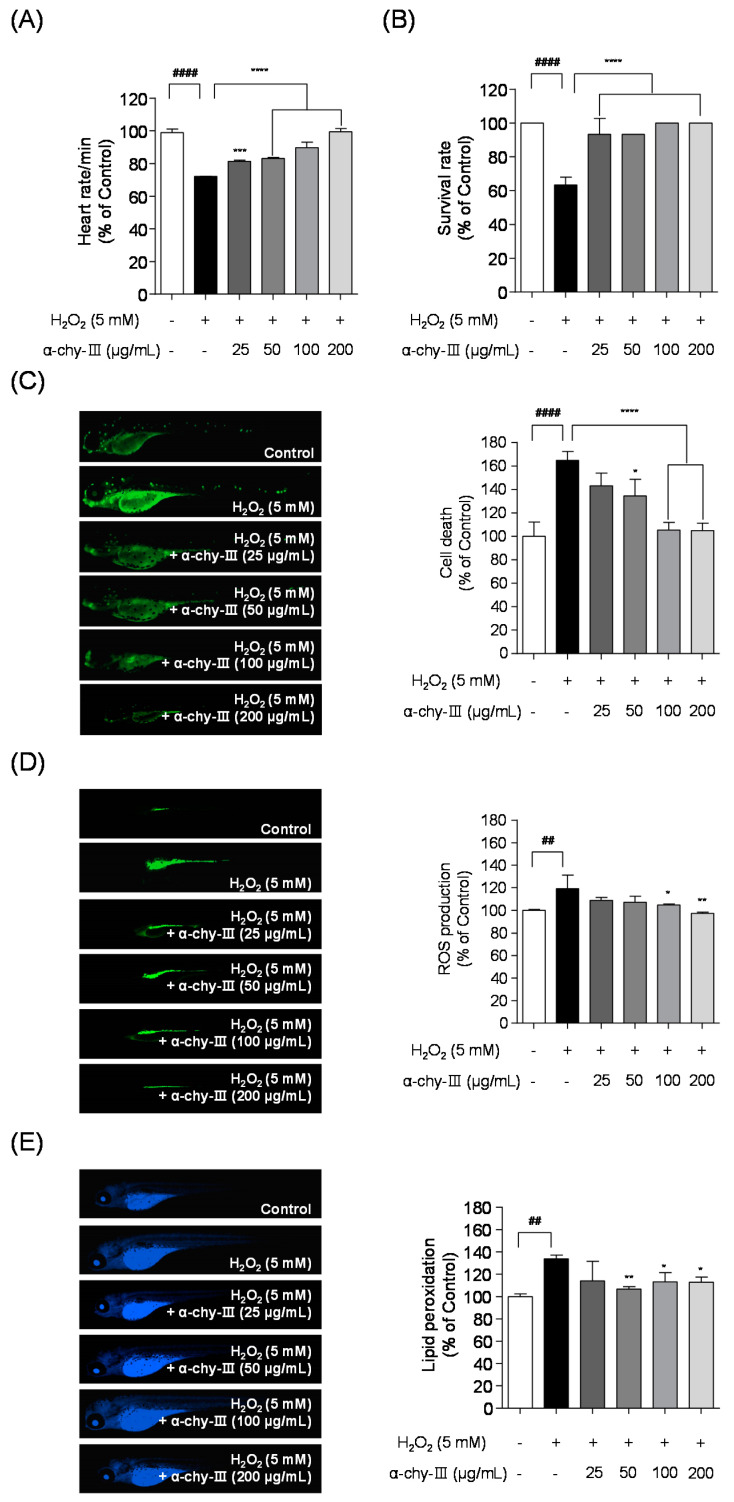
Effect of α-chy-III on H_2_O_2_ induced oxidative stress in survival rate (**A**), heart rate (**B**), cell death (**C**), ROS generation (**D**), and lipid peroxidation (**E**) in zebrafish embryos. Levels of fluorescence intensity were calculated using ImageJ software. Experiments were performed in triplicate and data are expressed as mean ± SD; Significant differences identified at * *p* < 0.05, ** *p* < 0.01, *** *p* < 0.001 and **** *p* < 0.0001 as compared to the H_2_O_2_ treated group; ^##^ *p* < 0.01 and ^####^ *p* < 0.0001 as compared to the control group. Statistical analyses were conducted using Tukey’s post hoc comparison and Duncan’s multiple range.

To establish the potential antioxidant activities of α-chy-III in an in vivo animal model, we measured the heart and survival rate, cell death, intracellular ROS production, and lipid peroxidation in H_2_O_2_ exposed zebrafish embryos. Figure 5A,B demonstrate that H_2_O_2_ significantly decreased both the heart rate (72.13 ± 0.11%) and survival rate (63.33 ± 4.71%). However, α-chy-III significantly improved both the heart rate and survival rate. As shown in Figure 5C–E, significant cell death (164.89 ± 7.62%), intracellular ROS (119.22 ± 11.89%), and lipid peroxidation (133.95 ± 3.28%) were observed in the H_2_O_2_ treated group compared with those in the control group. However, 200 µg/mL of α-chy-III significantly lowered cell death (105.01 ± 6.15%), intracellular ROS (97.17 ± 1.03%), and lipid peroxidation (112.84 ± 4.66%), respectively. These results indicated that α-chy-III has a protective effect against H_2_O_2_ induced cell death, intracellular ROS generation, and lipid peroxidation in vivo in zebrafish embryos.

## 4. Discussion

*Stichopus japonicus* (*S. japonicus*) contains various bioactive components, including proteins, polysaccharides, saponins, vitamins (A, C, B1, B2, and B3), and minerals (iron, zinc, calcium, and magnesium) [12]. Furthermore, *S. japonicus* is considered a healthy food in Asian countries [32,33]. In the present study, we aimed to separate the peptide fraction from *S. japonicus* and evaluate its in vivo antioxidant effects using a zebrafish animal model. In previous studies, the antioxidant activity of the low molecular weight hydrolysate fraction from *S. japonicas* was investigated at the in vitro levels [17,34,35]. However, the protective effect of the hydrolysate fraction of *S. japonicas* with low molecular weight has not been fully investigated in vitro and in vivo. Therefore, in the present study, we examined the protective effect of α-chy-III, which has a low molecular weight, against H_2_O_2_ induced oxidative stress in Vero cells and zebrafish embryos.

Recently, advanced techniques in zebrafish studies have developed the utility of zebrafish models in many research fields. Therefore, many researchers have used the zebrafish model as a predictive in vivo model for drug screening [36,37]. Zebrafish are used in the biotechnological field as a screening test because they possess many advantages, such as low cost, short generation time, genetic similarity, large number of eggs, and transparent embryos, to evaluate biological activity. Furthermore, the initial entry step of the in vivo study can easily measure heart rate and survival rate in zebrafish as a toxicity indicator of tested samples [38]. Furthermore, the heart rate and survival rate of zebrafish treated with tested samples can be used as indicators of toxicity [39]. Therefore, the zebrafish model is a popular animal model for drug screening or cytotoxic evaluation [40].

Marine animals are abundant sources of proteins or peptides that possess various biological activities, including antihypertensive, antioxidant, anticoagulant, and antimicrobial activities [41]. The bioactive peptide was obtained from the enzymatic hydrolysis of *S. japonicus*. In this study, enzyme-assisted hydrolysis was adopted considering the efficiency of the extraction techniques and the advantages of enzyme-assisted hydrolysis, which has been applied in the food and pharmaceutical industries [42]. Antioxidant activity was screened using ESR spectroscopy, which is a conventional analysis of the evaluation of free radical scavenging activity. Therefore, in many antioxidant studies, ESR spectroscopy was employed to assess potential antioxidative properties [43,44]. In the present study, we focused on the antioxidant effects of the SJH. The free radical scavenging activities of the SJH were screened, and their chemical compositions were analyzed. Among them, α-chymotrypsin assisted hydrolysate from *S. japonicus* (α-chy) showed the highest yield and significant hydroxyl radical scavenging activity (IC_50_ value, 1.03 ± 0.26). In addition, α-chy showed significant protection against H_2_O_2_ (1 mM) treatment. Therefore, it was selected as a candidate for further purification. In this study, we successfully separated the three different molecular weight fractions (>10 kDa; α-chy-I, 5–10 kDa; α-chy-II, < 5 kDa; α-chy-III) from α-chy through ultrafiltration (UF) and investigated their antioxidant properties. Among the UF fractions, the low molecular α-chy-III was found to have significant intracellular ROS inhibitory activity in H_2_O_2_ exposed Vero cells and strong protection against cell death, intracellular ROS generation, and lipid peroxidation in H_2_O_2_ exposed zebrafish embryos. These results corresponded with previous publications which reported potential antioxidant properties of enzymatic hydrolysates from silver carp muscle [45].

Excessive ROS generation by free radicals causes irreversible cell injuries such as cell membrane destruction, cell component oxidation, and DNA fragmentation, which may result in cell death and apoptosis [46]. We investigated whether α-chy-III has protective effects against apoptotic damage, including cell death, DNA fragmentation, and cell cycle arrest, in Vero cells under H_2_O_2_ induced oxidative stress. The fluorescent staining results indicated that α-chy-III administration markedly attenuated the H_2_O_2_ induced apoptotic cell death and DNA fragmentation. Furthermore, it could regulate apoptotic cell cycle arrest under oxidative stress conditions. These results were consistent with previous results from Thilina et al. (2020) [47]. 

The analysis of the amino acid composition demonstrated that α-chy-III is composed of 16 amino acids (aspartic acid, threonine, serine, glutamic acid, proline, glycine, alanine, valine, methionine, isoleucine, leucine, tyrosine, phenylalanine, histidine, lysine, and arginine). In particular, α-chy-III contained high levels of proline, glycine, methionine, tyrosine, phenylalanine, lysine, and arginine. According to previous publications, high levels of exogenous proline greatly reduce the diffusion of H_2_O_2_ and increase the activity of antioxidant enzymes [48]. The dietary sulfur-conjugated methionine showed excellent antioxidant capacity, and the supplementation of methionine increased antioxidant ability by stimulating antioxidant enzyme reaction [49]. Tyrosine residue performed important antioxidant functions by increasing the lipid density of cell membranes [50]. Lysine and glycine exhibited strong free radical scavenging activities against DPPH, hydroxyl, and alkyl radicals. Therefore, these results suggest that α-chy-III composed of the aforementioned active amino acids exhibits potential antioxidant capacities [51,52,53].

In summary, the α-chy significantly protected against H_2_O_2_ induced oxidative damage by suppressing intracellular ROS generation. In addition, its low molecular α-chy-III exerts potent antioxidant and anti-apoptotic effects under oxidative stress conditions via regulation of intracellular ROS generation and apoptotic DNA damage. Therefore, our findings suggest that α-chy and α-chy-III may be successfully utilized as a potent antioxidant ingredient in food and functional food industries.

## Figures and Tables

**Table 1:** Yields and chemical compositions of SJH.

Sample	Yield (%)	Proximate Composition (%)		
		Polysaccharide	Protein	Sulfate
DW	43.00 ± 0.01	3.18 ± 0.23	15.60 ± 0.69	7.41 ± 0.03
Al	42.50 ± 0.01	9.35 ± 2.98 ***	26.19 ± 1.04 ***	6.06 ± 0.04
α-chy	96.50 ± 0.06 ***	11.95 ± 2.07 ***	34.05 ± 0.97 ***	5.64 ± 0.08
Fla	68.50 ± 0.04 ***	9.35 ± 0.69 ***	36.15 ± 1.59 ***	6.58 ± 0.00
Koj	55.00 ± 0.02 ***	13.08 ± 5.51 ***	27.51 ± 1.66 ***	5.99 ± 0.17
Neu	55.50 ± 0.01 ***	13.08 ± 0.46 ***	31.41 ± 1.38 ***	5.55 ± 0.13
Pap	77.50 ± 0.05 ***	11.46 ± 0.46 ***	32.05 ± 1.45 ***	5.64 ± 0.08
Pep	62.50 ± 0.07 ***	8.21 ± 1.38	35.27 ± 2.97 ***	4.69 ± 0.08
Pro	78.50 ± 0.02 ***	11.46 ± 0.92 ***	28.58 ± 1.24 ***	5.58 ± 0.17
Try	82.00 ± 0.01 ***	10.64 ± 3.44 ***	36.24 ± 2.55 ***	6.14 ± 0.04

Alcalase; Al, α-chymotrypsin; α-chy, Flavourzyme; Fla, Kojizyme; Koj, Neutrase; Neu, Papain; Pap, Pepsin; Pep, Protamex; Pro, and Trypsin; Try; Significant difference identified at *** *p* < 0.001, as compared to the distilled water (DW) extract.

**Table 2:** Free radical scavenging activities of SJH.

Sample	Free Radical Scavenging Activity IC 50 Value, (mg/mL)		
	DPPH	Alkyl	Hydroxyl
DW	2.94 ± 0.06	0.39 ± 0.06	1.59 ± 0.02
Al	3.69 ± 0.04	0.38 ± 0.04	1.63 ± 0.18
α-chy	3.37 ± 0.19	0.39 ± 0.01	1.03 ± 0.26 *
Fla	4.40 ± 0.43	0.34 ± 0.02	1.40 ± 0.04
Koji	2.97 ± 0.51	0.44 ± 0.02	1.27 ± 0.09
Neu	5.31 ± 0.30	0.40 ± 0.02	2.96 ± 0.07
Pap	4.99 ± 0.00	0.40 ± 0.01	1.47 ± 0.08
Pep	3.89 ± 0.75	0.48 ± 0.03	3.46 ± 0.34
Pro	3.21 ± 0.42	0.42 ± 0.04	1.12 ± 0.07 *
Try	3.83 ± 0.14	0.38 ± 0.06	1.59 ± 0.01

Alcalase; Al, α-chymotrypsin; α-chy, Flavourzyme; Fla, Kojizyme; Koj, Neutrase; Neu, Papain; Pap, Pepsin; Pep, Protamex; Pro, and Trypsin; Try; Significant difference identified at * *p* < 0.05, as compared to the distilled water (DW) extract.

**Table 3:** Amino acid distribution in α-chy and its UF fractions.

Amino Acid	α-chy	α-chy-I	α-chy-II	α-chy-III
Aspartic acid	11.32	11.61	9.85	8.84
Threonine	5.40	5.29	4.92	4.55
Serine	5.35	4.96	4.93	4.81
Glutamic acid	16.91	15.91	15.96	14.54
Proline	1.79	8.53	9.75	9.79
Glycine	17.28	16.81	18.16	18.36
Alanine	8.01	6.93	7.46	7.03
Valine	3.97	4.04	3.04	2.87
Methionine	2.10	1.55	1.66	2.23
Isoleucine	3.30	3.42	2.62	2.50
Leucine	3.40	3.38	2.69	2.79
Tyrosine	3.06	2.15	2.89	3.60
Phenylalanine	3.39	2.46	2.83	3.60
Histidine	2.78	3.60	1.47	1.54
Lysine	3.60	2.78	3.48	4.10
Arginine	8.34	6.59	8.28	8.86
Total	100	100	100	100

## Data Availability

Not applicable.
